# Challenging lead extraction with quadripolar active fixation of coronary sinus with severe adhesion

**DOI:** 10.1016/j.hrcr.2023.06.005

**Published:** 2023-06-18

**Authors:** Yuhei Isonaga, Yukihiro Inamura, Akira Sato, Osamu Inaba, Masahiko Goya, Tetsuo Sasano

**Affiliations:** ∗ Department of Cardiology, Japanese Red Cross Saitama Hospital, Saitama, Japan; † Cardiovascular Center, International University of Health and Welfare, Mita Hospital, Tokyo, Japan; ‡ Department of Cardiovascular Medicine, Tokyo Medical and Dental University, Tokyo, Japan

**Keywords:** Case report, Attain Stability Quad, Active fixation lead, Lead extraction technique, Device infection

## Introduction

**Key Teaching Points**

• We experienced a case where the lead was difficult to remove using simple traction owing to severe adhesion between the coronary sinus (CS) and Attain Stability quadripolar lead (Medtronic plc, Dublin, Ireland).
• The severe adhesion was removed using a soft-tip guiding catheter to avoid damage to the CS, and the lead was successfully extracted.
• This technique may be useful in cases where extraction of the lead is inhibited owing to severe adhesion.

The efficacy of cardiac resynchronization therapy (CRT) for chronic heart failure is well established.^1^ The Attain Stability™ quadripolar (ASQ) 4798 lead (Medtronic plc, Dublin, Ireland) is an active fixation mechanism lead that provides flexibility in coronary sinus (CS) positioning. The side helix, which is spirally bonded to the surface of the lead, between its third and fourth poles, allows for more precise placement at any location and can be used in a wide variety of vessels. However, lead extraction may be difficult owing to severe adhesion of the helix site. In this report, we describe a case in which an ASQ lead that was difficult to extract using simple traction, owing to severe adhesion, was successfully extracted using an 8F guiding catheter.

## Case report

A 57-year-old male patient was admitted to our hospital with swelling at the device implantation site. The patient had a history of chronic heart failure (New York Heart Association class III) with severe left ventricular (LV) systolic dysfunction associated with hypertrophic cardiomyopathy in the diastolic phase, right bundle branch block with a QRS duration of 208 ms, first-degree atrioventricular block with a PR interval of 316 ms, and 4 prior ablations for atrial fibrillation/tachycardia. Eighteen months earlier, he had received the CRT-pacemaker Percepta™ (Medtronic plc). The right ventricular (RV) lead was placed in the RV apex septum by using an active fixation 5076 lead, and the right atrial (RA) lead was placed in the atrial septum by using a SelectSecure™ (Medtronic plc) lead, as no acceptable threshold site was left in the RA appendage owing to atrial remodeling. The ASQ lead was used as the LV lead and was fixed to the anterolateral branch of the CS.

One week prior to presentation, elastic soft swelling with no tenderness appeared near the puncture site of the subclavian vein, a short distance from the device. Laboratory test results showed elevated levels of inflammatory markers: C-reactive protein (4.96 mg/dL), white blood cell count (11,300/μL), and procalcitonin (1.7 ng/mL). Two sets of blood cultures were negative. A gallium-67 scan showed accumulation within the swollen area, suggesting the presence of active inflammation. Transthoracic echocardiography showed no evidence of infective endocarditis or vegetation in the leads. These findings strongly suggested local device infection, and device and lead extractions were performed.

**Figure 1:**
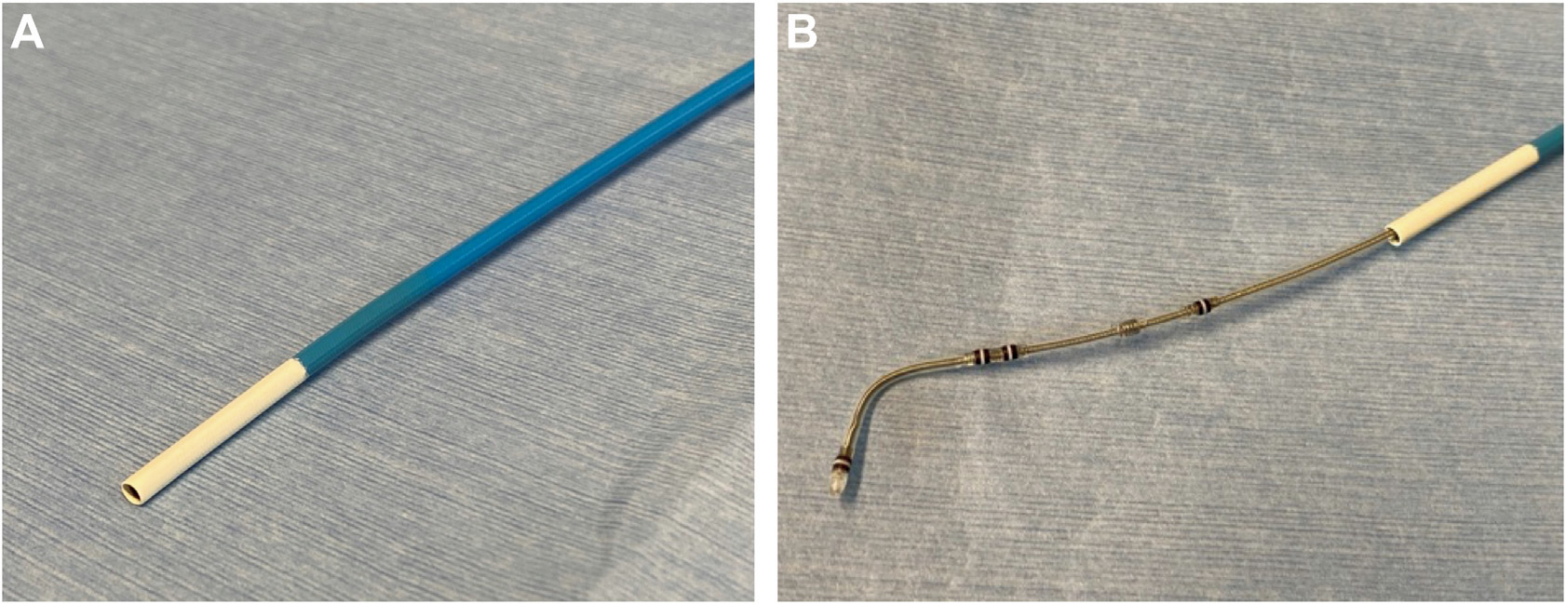
Guiding catheter used for carotid artery stenting to remove the adhesion. **A:** ROADMASTER RH8-ST1A (NIPRO, Tokyo, Japan). This catheter is a soft-tip, straight, 8F catheter. **B:** RH8-ST1A with Attain Stability quadripolar (ASQ) lead (Medtronic plc, Dublin, Ireland). The RH8-ST1A catheter is 2F larger than the ASQ lead diameter.

**Figure 2:**
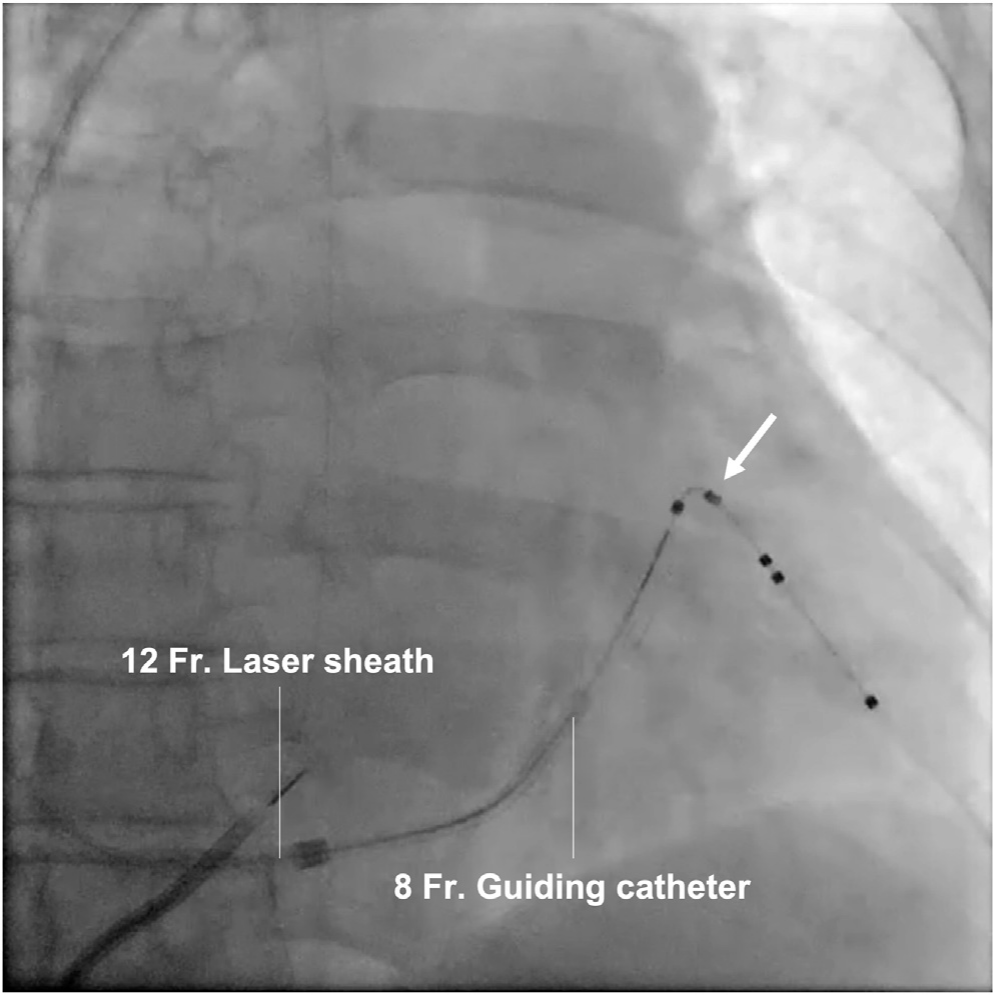
Fluoroscopy (anteroposterior view) demonstrating a 12F laser sheath and an 8F guiding catheter in the coronary sinus. The arrow shows the position of the side helix of the Attain Stability quadripolar 4798 lead (Medtronic plc, Dublin, Ireland).

**Figure 3:**
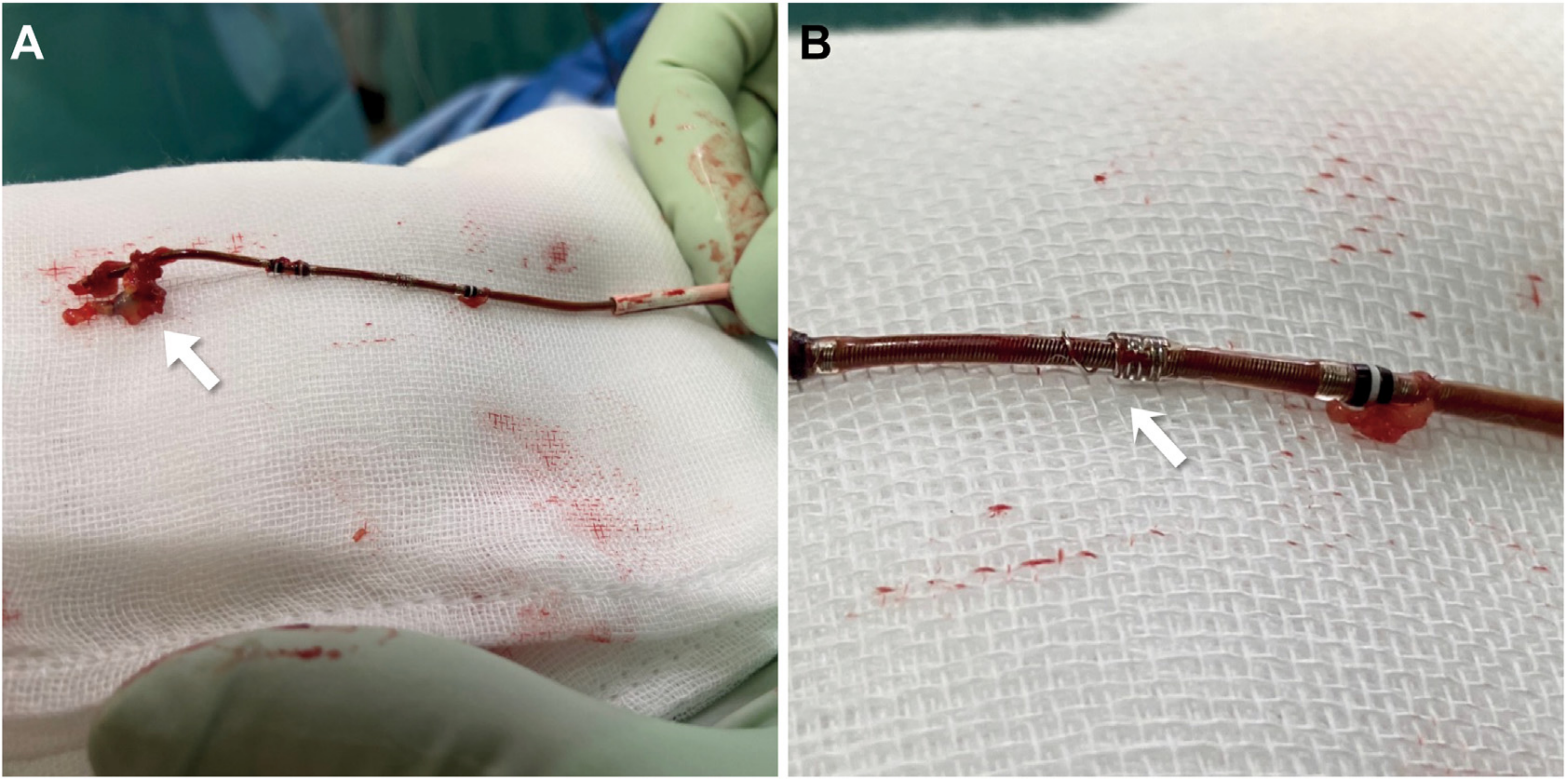
Lead and fixation screw after extraction. **A:** Attain Stability quadripolar (ASQ) 4798 lead (Medtronic plc, Dublin, Ireland) after extraction. The adhered fibrous tissue is compressed and adhered to the tip of the lead (*arrow*). **B:** Straightening of the active fixation screw of ASQ after extraction (*arrow*).

The extraction was performed in the operating room. General anesthesia was administered. The swollen area near the device was incised and the pus drained. The leads were disconnected from the device, and a standard stylet was inserted into the RV and LV leads. The active fixation helix of the RA and RV leads was retracted, and the leads were successfully extracted using simple traction. No rotation of the side helix was observed, despite the counterclockwise torque applied to the LV lead. Although movement of the fourth pole was confirmed by simple traction, severe adhesion was observed at the site of the side helix. Despite continuous strong traction, the LV lead resisted extraction, posing a high risk of CS branch injury. The LV lead was cut and a lead-locking device (LLD™ EZ; Philips, Amsterdam, Netherlands) was inserted, but insertion was only possible up to a point before the fourth pole, where it became locked. A 12F A GlideLight™ laser sheath (Philips) was used to advance the left subclavian vein using a laser, and the laser sheath with an outer sheath was delivered to the CS ostium. An 8F guiding catheter (ROADMASTER™ RH8-ST1A; NIPRO, Tokyo, Japan) was used for carotid artery stenting to remove the adhesion. This catheter was a soft-tip, straight catheter and 2F larger than the ASQ lead diameter to cover the fibrotic tissue that was expected to adhere to the ASQ lead (Figure 1). The guiding catheter was inserted into the laser sheath and advanced into the CS branch (Figure 2). As the guiding catheter was advanced, substantial resistance was encountered at the site of the side helix, but the adhesion was successfully removed (Supplemental Video 1). High resistance was also encountered between the second and third poles, but once the guiding catheter passed that point, the lead was simultaneously extracted (Online Supplemental Video 2). The side helix was straightened, and the compressed tissue was adhered to the head of the lead that was extracted (Figure 3).

Vancomycin (30 mg/kg/d) was intravenously administered for 2 weeks. Local infection was well controlled, and inflammatory marker levels were normalized. No perioperative complications occurred, and the patient was discharged on the fifth postoperative day.

This patient’s clinical and echocardiographic findings did not improve after CRT implantation (LV ejection fraction prior to implantation: 32%; after implantation: 29%); hence, he underwent CRT reprogramming optimization via echocardiography, but no improvement was achieved. An implantable cardioverter-defibrillator implantation for primary prevention was also considered after CRT removal, because the patient had reduced LV systolic function and a history of nonsustained ventricular tachycardia. However, the patient declined reimplantation and is being closely monitored.

## Discussion

We report here a case of an ASQ lead that was difficult to extract. The patient had an obvious local infection and was considered to have isolated pocket infection. Device removal and lead extraction were performed.

LV lead stability is necessary to improve the efficacy of CRT; however, stable implantation may be difficult owing to anatomical limitations. The ASQ lead demonstrated a high rate of successful implantation (96.8%) with a very low dislodgement rate (0.7%) at the 6-month follow-up in a large multinational study.^2^

However, active fixation leads may develop severe adhesion at the active fixation site, making extraction difficult. The StarFix™ (Medtronic plc) 4195 lead is an active fixation lead with a mechanism different from that of the ASQ lead. The StarFix lead has an expandable lobe, designed to hold the lead in place at the selected CS branch position. Extraction of the StarFix lead is challenging because of the impossibility of undeploying the lobe, and may be even more difficult because of frequently observed venous obstruction around the fixation mechanism.^3^ The StarFix lead is not currently marketed, and experience with its extraction is limited to a small number of patients and operators.

Reports on the extraction of long-term implanted Attain Stability leads are scarce. In a sheep model with an Attain Stability bipolar 20066 lead (Medtronic plc), implanted leads were successfully extracted with a manual traction force of less than 1 kg up to 2 years after implantation, with little resultant fibrosis around the helix.^4^ Among the small number of reports of Attain Stability lead extraction, the longest lead implantation period was 63 months.^5^, ^6^, ^7^, ^8^, ^9^, ^10^ In that report, the bipolar lead was easily and safely removed by simple traction using a standard stylet without using an additional tool.^10^ We are aware of only 1 previous report of ASQ lead extraction.^11^ In the report, the period from implantation was 16 months and the ASQ lead could be extracted with simple manual traction, although severe adhesion was observed at the helix site. Based on these reports, we initially assumed that simple traction would be sufficient for extraction; however, the procedure proved to be considerably challenging. To our knowledge, this is the first report on a difficult case requiring an extra tool for ASQ lead extraction. The possibility of difficulties in ASQ lead extraction should be considered prior to implantation in younger patients and patients at elevated risk of infection.

## Conclusion

We encountered a case in which extraction of an ASQ lead was difficult because of severe adhesion at the active fixation site 18 months after implantation. Simple traction was insufficient to extract the lead, and a guiding catheter was useful to remove the adhesion around the helix site. The use of an ASQ lead should be carefully considered to avoid difficulties in lead extraction.

## Appendix Supplementary Data

Supplemental Video 1 Although there was substantial resistance at the site of the side helix, the adhesions were successfully removed with guiding catheter.

Supplemental Video 2 There was also high resistance between the second and third poles. Guiding catheters removed the adhesions, and the lead was simultaneously extracted.
