# Supplementary material for: Challenging lead extraction with quadripolar active fixation of coronary sinus with severe adhesion
Source: HeartRhythm Case Rep. 2023 Jun 18;9(9):614–7. doi: 10.1016/j.hrcr.2023.06.005 (PMC10511931; doi:10.1016/j.hrcr.2023.06.005)
Supplement: Supplementary Video Legends [file mmc3.docx]

**Supplementary Video legends**

Supplementary Video S1

Although there was substantial resistance at the site of the side helix, the adhesions were successfully removed with guiding catheter.

Supplementary Video S2

There was also high resistance between the second and third poles. Guiding catheters removed the adhesions, and the lead was simultaneously extracted.
